# Adenocarcinoma of the third portion of the duodenum in a man with CREST syndrome

**DOI:** 10.1186/1477-7819-6-106

**Published:** 2008-10-01

**Authors:** Georgios Anastasopoulos, Athanasios Marinis, Christos Konstantinidis, Theodosios Theodosopoulos, Georgios Fragulidis, Ioannis Vassiliou

**Affiliations:** 1 Second Department of Surgery, Areteion University Hospital, 76 Vassilisis Sofia's Ave, 11528, Athens, Greece

## Abstract

**Background:**

CREST (Calcinosis, Raynaud's phenomenon, Esophageal dysmotility, Sclerodactyly and Telangiectasias) syndrome has been rarely associated with other malignancies (lung, esophagus).This is the first report of a primary adenocarcinoma of the third portion of the duodenum in a patient with CREST syndrome.

**Case presentation:**

A 54-year-old male patient with CREST syndrome presented with colicky postprandial pain of the upper abdomen, diminished food uptake and a 6-Kg-body weight loss during the previous 2 months. An ulcerative lesion in the third portion of the duodenum was revealed during duodenoscopy, with a diagnosis of adenocarcinoma on biopsy specimen histology. The patient underwent a partial pancreatoduodenectomy. No adjuvant therapy was instituted and follow-up is negative for local recurrence or metastases 21 months postoperatively.

**Conclusion:**

CREST syndrome has been associated with colon cancer, gastric polyps, familial adenomatous polyposis (FAP) syndrome and Crohn's disease; however, this is the first report of a primary adenocarcinoma of the duodenum in a patient with CREST syndrome. However, any etiologic relationship remains to be further investigated.

## Background

CREST syndrome (Calcinosis, Raynaud's phenomenon, Esophageal dysmotility, Sclerodactyly and Telangiectasias) has been rarely associated with other malignancies (lung, esophagus), whereas duodenal adenocarcinoma has never been reported to be associated with this subtype of systemic sclerosis [1-3]. This is the first report of a primary adenocarcinoma of the third portion of the duodenum in an adult male patient with CREST syndrome.

## Case presentation

**Figure 1:**
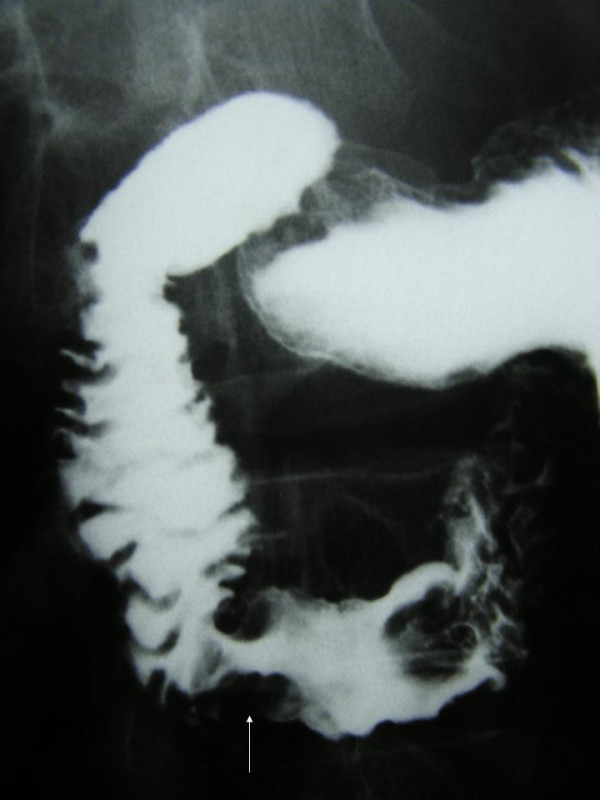
Barium upper GI series showing a stricture (arrow) in the transition of the second to the third portion of the duodenum.

**Figure 2:**
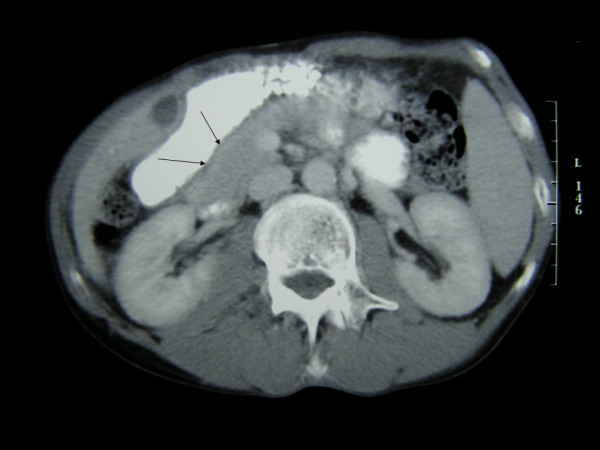
Computed tomography of the abdomen depicting a mass (arrows) in the duodenum.

A 54-year-old man was referred to our clinic because of colicky postprandial pain of the upper abdomen during the last month, with aggravation 5 days before admission. The patient had diminished the amount of food uptake and had also a 6-Kg-body weight loss during the previous 2 months. His past medical history included CREST syndrome under cortisone therapy during the last year and primary repair of the left ureter and the small intestine due to traumatic perforation in a car accident 17 years ago. Physical examination revealed a pale, malnourished patient and an unremarkable abdomen without tenderness or distension. Besides an anemia, the other blood investigations, as well as tumor markers, were within normal limits. Plain abdominal radiographs showed no pathological findings, while contrast-enhanced series demonstrated a stricture in the transition area from the second to the third portion of the duodenum (Figure 1). Abdominal computed tomography showed a mass in the duodenum, without evidence of metastases (Figure 2). Thorax computed tomography was negative for metastases as well. Gastroduodenoscopy revealed an ulcerated lesion between the second and third portion of the duodenum and the biopsies showed an adenocarcinoma. Colonoscopy was normal.

Laparotomy, through a bilateral subcostal incision, revealed a large tumor locally invading the third portion of the duodenum and possibly the pancreas. Therefore, a partial pancreatoduodenectomy (Whipple's procedure) was carried out. Histological examination revealed a low-grade duodenal adenocarcinoma of maximal diameter 4 cm, which infiltrated the duodenal wall and the fibro-adipose tissue of the mesentery. Metastasis was found in one out of ten lymph nodes examined, while the surgical margins were negative for microscopic disease.

The patient's postoperative course was complicated with a peripancreatic-remnant collection and a lower respiratory infection, which were treated conservatively.

No additional therapy was instituted and 21 months after the operation the patient is alive, without any evidence of local recurrence or metastases.

## Discussion

Primary duodenal adenocarcinoma represents a relatively rare clinical entity. Duodenum constitutes the most predominant site (55%) of occurrence of adenocarcinoma throughout the small intestine, followed by the jejunum (18%) and ileum (13%) [4]. The distribution pattern of adenocarcinoma within the duodenum is reported to parallel the length of each portion. The first portion is affected in 15%, the second portion in 40% and the third and fourth portion in 45% of the cases [5].

Most of these neoplasms are asymptomatic until they become large in size. Partial duodenal obstruction, with associated symptoms of crumpy postprandial pain of the upper abdomen, nausea and vomiting, is the most common mode of presentation. Hemorrhage, usually indolent, is the second most common mode of presentation. Due to their location these tumors cause non-specific and vague symptoms leading to a delay of diagnosis [6]. Periampullary tumors may cause obstructive jaundice or pancreatitis.

Barium upper GI series and endoscopy of the upper gastrointestinal (GI) tract establish the diagnosis with a sensitivity of 81,9% and 88%, respectively [7], while colonoscopy and thoraco-abdominal computed tomography are carried out to exclude any synchronous or metastatic cancer.

The only possibility of cure is obtained by a radical resection. The percentage of resectability with a curative intent is reported to range from 50% to 73% [7]. For the tumors located in the first and the second portion of the duodenum, a Whipple's procedure is considered necessary for the radical resection of the tumor and its lymphatic drainage. For tumors of the third and fourth portion, a segmental resection of the duodenum with the appropriate lymphadenectomy is performed with a curative intent [8]. Chemotherapy and radiation are considered to have only a small contribution to the overall survival or the disease-free survival of these patients [9].

Primary duodenal adenocarcinoma is an aggressive tumor with an overall 5-year survival rate of about 25% [7], which can be significantly improved up to 54% after curative resection [8]. The most important prognostic factors are the stage of the disease and the location of the tumor. Tumors with negative margins in the surgical specimen, which are located in the first and second portions of the duodenum, are considered to have a better prognosis. Other series report that histologic grade, depth of invasion, tumor size and metastases to regional lymph nodes influence the survival rate [7-10].

Primary duodenal adenocarcinoma has been associated with colon cancer, gastric polyps, especially villous and tubulovillous adenomas, familial adenomatous polyposis (FAP) and Crohn's disease. It has been stated that primary duodenal adenocarcinoma is one of the main causes of death in patients with FAP [11]. A case of an early duodenal adenocarcinoma from a Brunner's gland has been reported [12]. Another case of primary duodenal adenocarcinoma in a patient with neurofibromatosis type 1 has also been reported [13].

Limited cutaneous scleroderma or CREST syndrome consists one of the two main subsets of systemic sclerosis, which is a multisystemic disorder of unclear pathogenesis, characterized by inflammatory, vascular and fibrotic changes of the skin and various internal organ systems (GI tract, lungs, heart and kidneys) and involves immunologic mechanisms leading to vascular endothelial damage and fibroblast activation. CREST syndrome consists of calcinosis, Raynaud's phenomenon, esophageal dysmotility, sclerodactyly and telangiectasias and is associated with a better prognosis [14].

The association of this syndrome with malignancies is extremely rare. A case of CREST syndrome and adenocarcinoma of the lung has been reported [1]. It is also suggested that patients with scleroderma and Barrett's metaplasia have an increased risk of complications, such as strictures or adenocarcinoma [2]. Another case of progressive systemic sclerosis (CREST syndrome), sarcoidosis and esophageal adenocarcinoma in a 50-year-old Japanese female has been reported [3]. Finally, we report the first case of a male patient with CREST syndrome and duodenal adenocarcinoma.

## Conclusion

A relationship between duodenal adenocarcinoma and limited cutaneous scleroderma has not yet been described and in our case is considered a matter of coincidence. Whether underlying mechanisms exist (immunologic, genetic, etc) remains to be elucidated with further research.

## Competing interests

The authors declare that they have no competing interests.

## Authors' contributions

GA, IV and CK carried out the surgical procedure and contributed to the design of the study; GA and AM gathered the data, drafted the manuscript and critically revised it; IV, TT and GF revised and finally approved the manuscript for been published. All authors read and approved the final manuscript.

## Consent

Written informed consent was obtained from the patient for publication of this case report and any accompanying images. A copy of the written consent is available for review by the Editor-in-Chief of this journal.
